# The Cellular DExD/H-Box RNA-Helicases UAP56 and URH49 Exhibit a CRM1-Independent Nucleocytoplasmic Shuttling Activity

**DOI:** 10.1371/journal.pone.0022671

**Published:** 2011-07-22

**Authors:** Marco Thomas, Peter Lischka, Regina Müller, Thomas Stamminger

**Affiliations:** Institute for Clinical and Molecular Virology, University of Erlangen-Nuremberg, Erlangen, Germany; Chinese University of Hong Kong, Hong Kong

## Abstract

Cellular DExD/H-box RNA-helicases perform essential functions during mRNA biogenesis. The closely related human proteins UAP56 and URH49 are members of this protein family and play an essential role for cellular mRNA export by recruiting the adaptor protein REF to spliced and unspliced mRNAs. In order to gain insight into their mode of action, we aimed to characterize these RNA-helicases in more detail. Here, we demonstrate that UAP56 and URH49 exhibit an intrinsic CRM1-independent nucleocytoplasmic shuttling activity. Extensive mapping studies identified distinct regions within UAP56 or URH49 required for (i) intranuclear localization (UAP56 aa81-381) and (ii) interaction with REF (UAP56 aa51-428). Moreover, the region conferring nucleocytoplasmic shuttling activity was mapped to the C-terminus of UAP56, comprising the amino acids 195-428. Interestingly, this region coincides with a domain within Uap56p of *S. pombe* that has been reported to be required for both Rae1p-interaction and nucleocytoplasmic shuttling. However, in contrast to this finding we report that human UAP56 shuttles independently from Rae1. In summary, our results reveal nucleocytoplasmic shuttling as a conserved feature of yeast and human UAP56, while their export receptor seems to have diverged during evolution.

## Introduction

DExD/H-box RNA-helicases are implicated in virtually every step of RNA metabolism, including transcription, splicing, mRNA export, ribosome biogenesis, translation initiation or mRNA degradation [1]–[3]. One member of this family of RNA-helicases is UAP56 (alternatively named BAT1 in human, HEL in *Drosophila* or Sub2p in yeast), a protein that has both RNA-stimulated ATP-binding/hydrolysis activity and ATP-dependent RNA unwinding activity [4]–[6]. Initially, UAP56 was identified as a U2AF^65^-associated protein, involved in mRNA splicing [4]. Most importantly, however, it has been demonstrated that UAP56 also fulfills a key function for cellular mRNA export by interacting with the cellular adaptor protein REF [7], [8]. The RNA export factor REF shuttles between the nucleus and the cytoplasm and bridges mRNAs to the major mRNA transport receptor TAP by binding directly to both macromolecules [9]–[11]. Interestingly, UAP56 is able to recruit REF to intron-containing as well as to intron-free pre-mRNAs. In the first of these two scenarios, UAP56 is a constituent of the exon junction complex (EJC) and thereby couples splicing and mRNA export [12], [13]. In the second setting, UAP56, as a component of the TREX complex, couples RNA-polymerase II-dependent transcription elongation with mRNA export [14], [15]. In either case, REF recruitment is followed by formation of a ternary complex and upon methylation of REF, mRNA is transferred to TAP [16], [17]. Current models assume that UAP56 leaves the mRNP complex in the nucleus, since REF interacts either with TAP or UAP56 in a mutually exclusive manner [15], [18]. Since REF is dispensable in *Drosophila* [19], it is conceivable that additional adapter proteins are also able to transfer mRNAs to TAP and, hence, facilitate mRNA export. Indeed, the shuttling human SR proteins 9G8, SRp20 and SF2/ASF directly bind to TAP via their short arginine-rich peptides and function as export factors [20]–[22].

**Figure 1:**
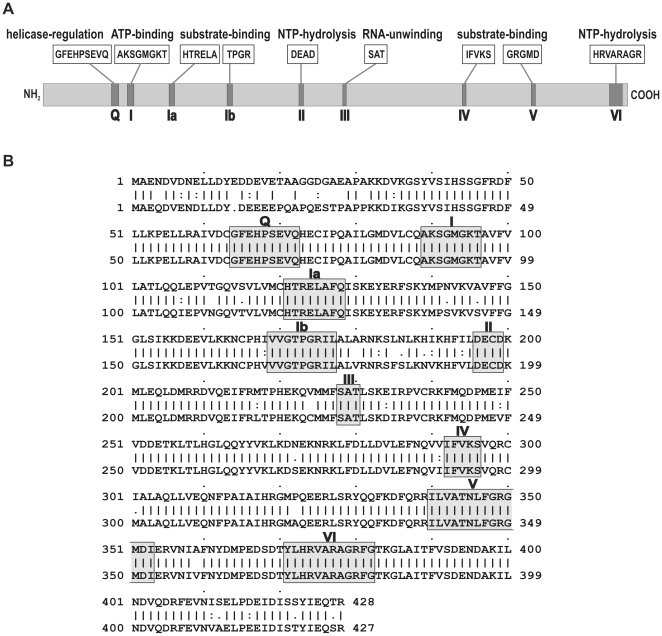
DExD/H-box RNA-helicases UAP56 and URH49: (A) Schematic representation of a DExD/H-box RNA helicase. The conserved helicase motifs are indicated by shaded boxes and roman numerals. The known or supposed function of the respective DExD/H-box motif, along with its consensus amino acid sequence is highlighted above the protein. (B) Alignment of the 428 amino acids of UAP56 (upper strand) and the 427 amino acids of URH49 (lower strand). The above mentioned DExD/H-box RNA-helicase motifs are indicated by shaded boxes with their respective roman numeral.

While only one gene corresponding to UAP56 exists in *Saccharomyces cerevisiae* and *Drosophila melanogaster*, an additional UAP56 related helicase termed URH49 (alternatively DDX39) has been discovered in humans [23]. The URH49 protein shares 90% amino acid sequence homology with UAP56 although it is 100% conserved within the DExD/H-box RNA-helicase motifs and shows the highest degree of divergence within its N-terminus (Fig. 1). Consequently, URH49 as well as UAP56 (i) are able to complement a Sub2p deletion and (ii) interact with the adaptor protein REF in yeast, suggesting that both proteins exert similar or redundant functions in mammalian cells [23].

Given the diverse functions of UAP56/URH49 during splicing and mRNA export it is not surprising that these proteins are essential in yeast, *Drosophila*, *Caenorhabditis elegans* or human cells [7], [15], [24], [25]. Moreover, the functional importance of these two RNA-helicases has been further sustained by our observation that mRNA-export of human cytomegalovirus, a ubiquitous herpesvirus, is dependent on UAP56/URH49-recruitment by a multifunctional viral protein termed pUL69 [26]. In order to gain further insight into cellular and/or viral mRNA metabolism, we conducted this work to characterize UAP56/URH49 in more detail.

Here, we report that the human DExD/H-box RNA-helicases UAP56 and URH49 exhibit an unexpected nucleocytoplasmic shuttling activity that is mediated by a CRM1-independent export signal. While UAP56p of *Schizosaccharomyces pombe* shuttles by recruitment of Rae1p, we report that human UAP56 exerts a Rae1-independent shuttling activity. Intriguingly, our data suggest a so far uncharacterized additional cytoplasmic function of UAP56 and URH49.

## Results

### UAP56 and URH49 exert nucleocytoplasmic shuttling activity

During the past decade an accumulating line of evidence suggested that, in addition to REF, several other proteins such as the SR proteins 9G8, SRp20 and SF2/ASF are capable to transfer mRNAs to the heterodimeric export receptor TAP/p15 in order to facilitate their translocation to the cytoplasm [10], [11], [20]–[22]. Interestingly, most of these adapter proteins possess an intrinsic nucleocytoplasmic shuttling activity. As the essential RNA-helicases UAP56 and URH49 are also involved in mRNA export, it was tempting to analyze whether these proteins also shuttle between the nucleus and the cytoplasm.

**Figure 2:**
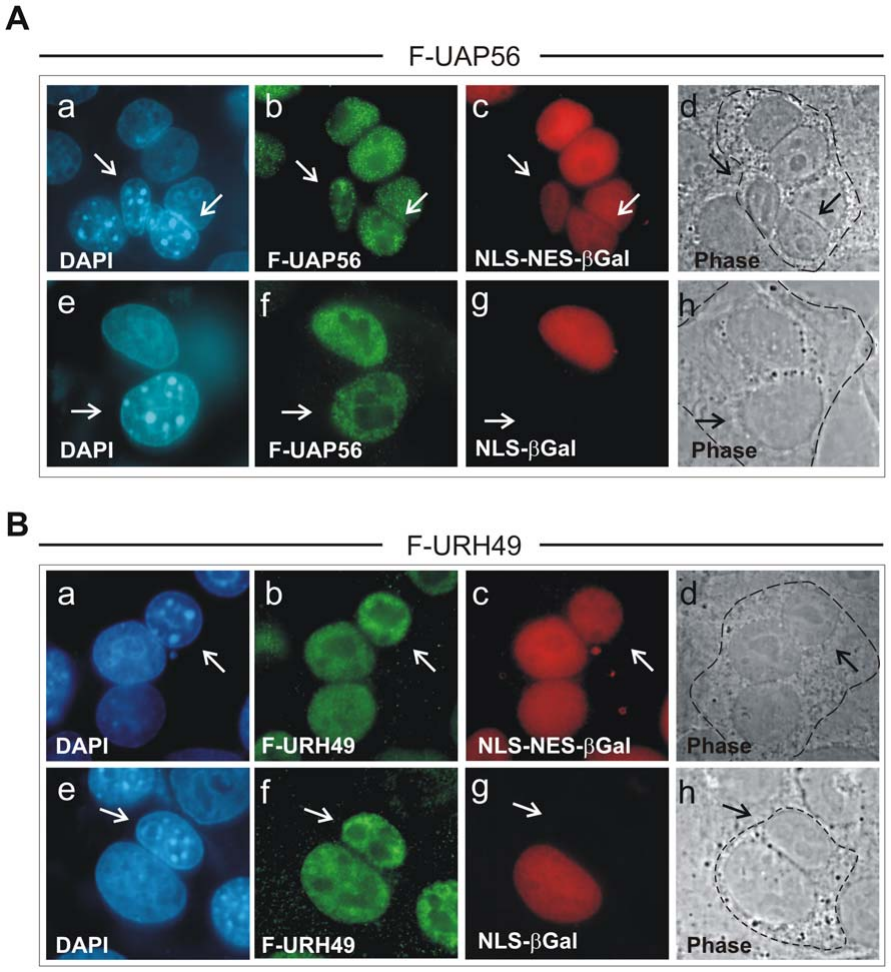
Nucleocytoplasmic shuttling of UAP56 (A) and URH49 (B) in transfected HeLa cells. HeLa cells were cotransfected with expression plasmids for FLAG-UAP56 (panel A) or FLAG-URH49 (panel B) and one of the internal control plasmids as indicated (a–d, + CFNrev-βGal; e–f, + CFN-βGal). The transfected cells were subjected to an interspecies heterokaryon assay as described in the Materials and Methods section. In order to detect and discriminate the respective proteins, double immunofluorescence analysis was performed using polyclonal anti-FLAG serum (b and f) and monoclonal anti-β-galactosidase antibody (c and g). DAPI, counterstaining of the nuclei (a and e); Phase, phase contrast image of the heterokaryon (d and h); murine nuclei are indicated by arrows.

First, we investigated whether UAP56 and URH49 are shuttling in the context of mammalian cells. To test this, HeLa cells were cotransfected with an expression plasmid for FLAG-UAP56 (Fig. 2A) or FLAG-URH49 (Fig. 2B) and one of the internal control plasmids CFN-βGal or CFNrev-βGal. CFNrev-βGal encodes β-galactosidase (βGal) fused to the NLS of the SV40 T-antigen and the NES of the HIV-1 Rev protein. This fusion protein thereby served as a positive internal control as it shuttles between the nucleus and the cytoplasm in transfected cells. CFN-βGal expresses only the SV40 T-antigen NLS fused to βGal and is therefore restricted to nuclei, hence serving as an internal negative control [27]. Transfected cells were allowed to synthesize FLAG-UAP56 or -URH49, in combination with one of the control proteins. The next day transfected HeLa cells were cocultivated with mouse NIH3T3 cells for another 24h until heterokaryon formation was induced as described in Materials and Methods. After fixation, indirect immunofluorescence analyses were performed using antibodies directed against the FLAG-tag and βGal. In interspecies heterokaryons that coexpressed FLAG-UAP56 and NLS-NES-βGal (as encoded by CFNrev-βGal), both proteins were observed in murine and human nuclei (Figure 2A, a–d). In contrast, when the localization of FLAG-UAP56 and NLS-βGal (as encoded by CFN-βGal) was assessed, only FLAG-UAP56 was found to be present in murine nuclei, while NLS-βGal was detected exclusively in human nuclei (Fig. 2A, e–h). Analogous results were obtained in interspecies heterokaryons that coexpressed FLAG-URH49 and NLS-NES-βGal (Figure 2B, a–d) or FLAG-URH49 and NLS-βGal (Figure 2B, e–h). In summary, the results presented here demonstrate that both UAP56 and URH49 exhibit a nucleocytoplasmic shuttling activity in mammalian cells.

### CRM1-independent shuttling of UAP56/URH49

**Figure 3:**
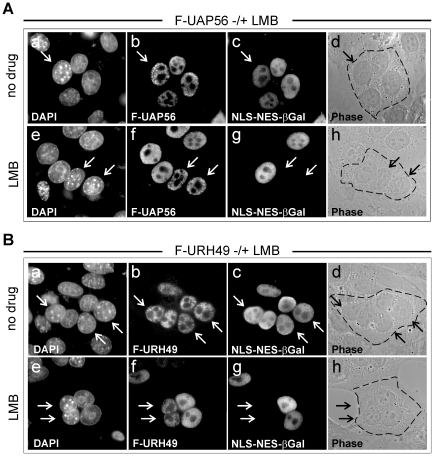
CRM1-independent shuttling activity of UAP56 (A) and URH49 (B). (A+B) Heterokaryon assay: Effect of LMB on shuttling of FLAG-UAP56 or FLAG-URH49 as determined by interspecies heterokaryon analysis. HeLa cells were cotransfected with expression constructs for FLAG-UAP56 (A) or FLAG-URH49 (B) in combination with the plasmid CFNrev-βGal. Three hours prior to fusion and throughout the experiment, cells were incubated in the absence (a–d) or presence of 2.5 ng/ml leptomycin B (LMB; panels e–h). Two hours after fusion, proteins were detected by indirect immunofluorescence analyses essentially as described in the legend of Figure 2. Murine nuclei are indicated by arrows.

It is well established that nucleocytoplasmic shuttling proteins utilize specific nuclear export signals (NESs) in order to be transported to the cytoplasm. The vast majority of shuttling proteins comprise an NES that is similar to the leucine-rich NES of the HIV-1 Rev protein. The cellular export receptor targeted by such an NES is called CRM1 and can specifically be blocked by the antibiotic leptomycin B (LMB) [28]–[31]. However, using the NetNES1.1 server (www.cbs.dtu.dk/services/) for *in silico* prediction, we were neither able to detect a leucine-rich NES in UAP56 nor in URH49. This suggested that UAP56/URH49-export is either mediated by a non-conventional, but CRM1-dependent NES or by a CRM1-independent NES. To address this question, FLAG-UAP56 or FLAG-URH49 transfected HeLa cells were treated with the CRM1-specific inhibitor LMB (Fig. 3A and B, panels e–h) or kept untreated (panels a–d) and heterokaryon assays were performed as described above. As expected, LMB inhibited nucleocytoplasmic shuttling of the cotransfected NLS-NES-βGal protein containing the HIV-1 Rev-NES (Fig. 3A and B, panels g), while, in contrast, shuttling of FLAG-UAP56 or FLAG-URH49 was completely unaffected by LMB (Fig. 3A and B, panels c). These findings indicate that both proteins are not transported via the CRM1-export pathway.

### Delineation of domains within UAP56 required for nuclear localization

**Figure 4:**
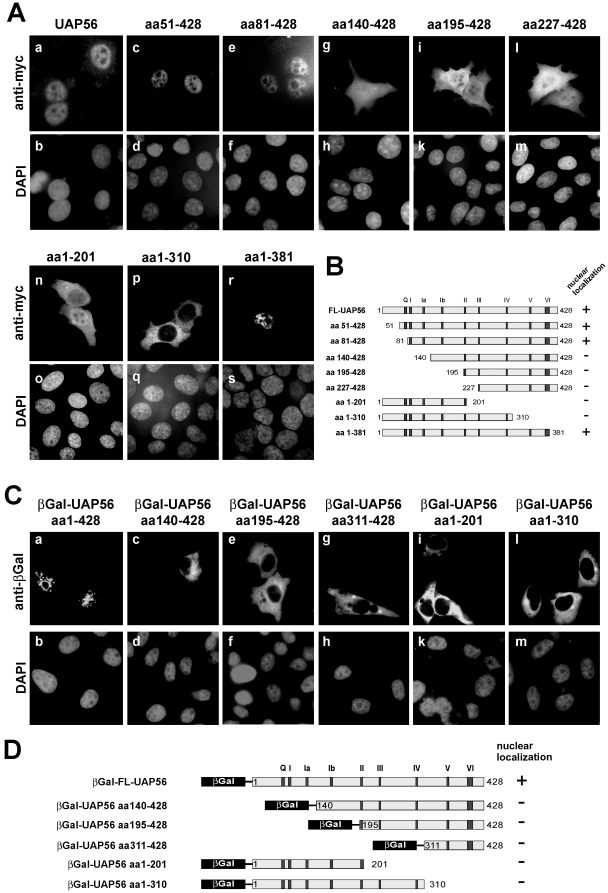
Intracellular localization of UAP56-truncation mutants. (A) Indirect immunofluorescence analyses: HeLa cells were transfected with expression plasmids encoding myc-tagged full-length UAP56 (panels a and b), N- (panels c–m) or C-terminally (panels n–s) truncated UAP56 as indicated. Two days posttransfection cells were fixed and subjected to indirect immunofluorescence analyses using a monoclonal antibody directed against the myc-tag. DAPI, counterstaining of cell nuclei. (B) Schematic representation of the constructs used in (A). (C) Subcellular localization of UAP56 (full-length and truncated) in fusion with the SV40 NLS and β-galactosidase: HeLa cells were transfected with the indicated expression plasmids followed by indirect immunofluorescence analyses using a monoclonal antibody against β-galactosidase (anti-βGal). (D) Schematic representation of the constructs used in (B). Nuclear localization of the respective construct is indicated by a ‘+’, while cytoplasmic localization is indicated by a ‘-‘.

While these experiments demonstrated a nuclear export activity of UAP56 and URH49, it remained to be elucidated how these proteins are imported into the nucleus. Although *in silico* predictions did not detect a classical NLS within UAP56 and URH49 we and others demonstrated a nuclear localization of both proteins [26], [32]. To uncover domains required for nuclear localization we sought to determine the intracellular distribution of a series of N- or C-terminally truncated UAP56 variants that were fused to the myc-epitope. HeLa cells were transfected with the respective expression plasmids and the subcellular localization of transiently expressed proteins was assessed by indirect immunofluorescence analysis using a monoclonal anti-myc antibody. As illustrated by Fig. 4 A and B, N-terminal truncation of UAP56 beyond amino acid 81 (panels g and h) and C-terminal truncation beyond amino acid 381 (panels p and q) abrogated nuclear localization. In order to investigate whether the partial nuclear localization of truncated UAP56 variants could be due to passive diffusion into the nucleus (see Fig 4, panels g–o), we constructed in-frame fusions with the open reading frame coding for the large cytoplasmic protein β-galactosidase (βGal). As shown in Fig. 4 C and D, all truncated βGal-ΔUAP56 fusion proteins exhibited an exclusive cytoplasmic localization, while a fusion of full-length UAP56 with βGal was nuclear. Therefore, we conclude that amino acids 81–381 of UAP56 are required for proper nuclear localization of this RNA helicase.

### Delineation of the pUL69- and REF-interaction motifs in UAP56/URH49

**Figure 5:**
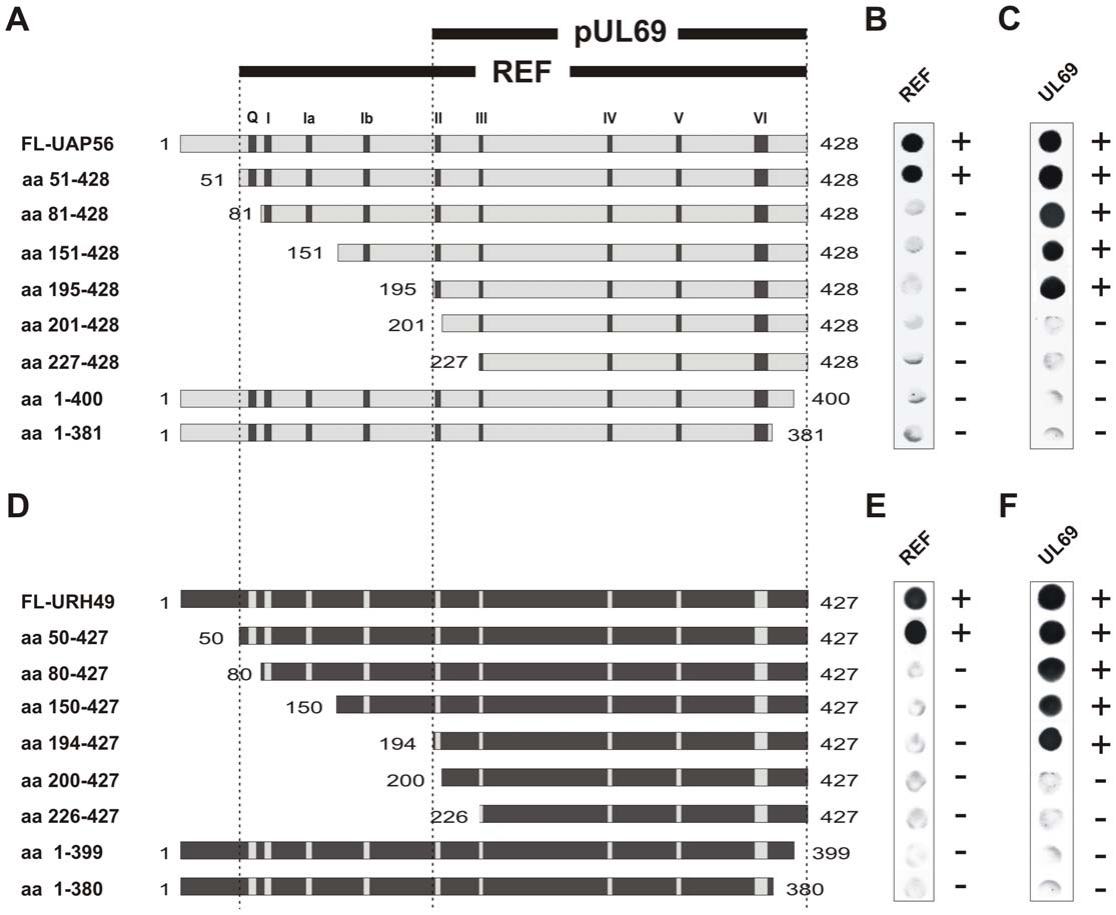
Mapping of the REF- and pUL69-interaction domain within UAP56 and URH49. (A–F) Yeast two-hybrid analysis: Yeast cells were transformed with full-length, or N- and C-terminal deletion mutants of UAP56 (A) or URH49 (D) as in-frame fusions to the GAL4 activation domain in combination with a vector encoding REF (B+E) or UL69 (C+F) in fusion with the GAL4 DNA-binding domain as indicated. Growth-selected yeast cells were analyzed in filter-lift assays for the expression of β-Gal, indicated by blue-staining of the respective colony. The mapped interaction regions that were required and sufficient for pUL69- (aa195/194-428/427) or REF-interaction (aa51/50-428/427) of UAP56 or URH49, respectively, are highlighted by black bars.

Taking into account that UAP56 and URH49 exhibit a complex secondary structure (see Fig. 1), we were interested to analyze whether the association with known protein interaction partners could be mapped to domains that differ from the nuclear localization domain. In HCMV-infected cells UAP56 or URH49 are constituents of multiprotein complexes containing cellular REF and the cytomegaloviral protein pUL69 (see Introduction) [26]. Thus, we started to delineate the domains within UAP56/URH49 required for interaction with these proteins by yeast two-hybrid analyses as reported before [33]. Briefly, *Saccharomyces cerevisiae* cells (strain Y153) were transformed with a combination of vectors encoding truncated versions of UAP56 or URH49 in fusion with the GAL4-activation domain (Fig. 5 A and D, as indicated) in combination with either REF (Fig. 5 B and E) or pUL69 (Fig. 5 C and F) fused to the GAL4 DNA-binding domain. Growth-selected yeast cells were then analyzed by filter-lift assays for expression of the β-galactosidase reporter gene. As depicted in figure 5, only the very N-terminal 51/50 amino acids of UAP56 or URH49, respectively, could be deleted without abrogating the interaction with REF (see panels B and E). In contrast, a smaller C-terminal domain of both proteins comprising the amino acids 195–428 of UAP56 or 194–427 of URH49, respectively, was sufficient for interaction with the viral protein pUL69 (see panels C and F). Consequently, these results imply that different domains within UAP56/URH49 were required for interaction with REF or pUL69.

**Figure 6:**
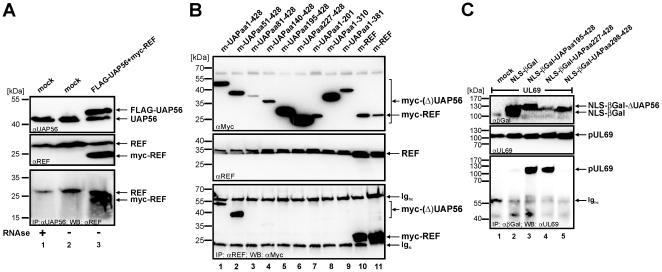
Confirmation of UAP56 interaction domains in mammalian cells. Coimmunoprecipitation analyses to confirm the interaction of endogenous UAP56 and REF (A) and to map the UAP56-domains required for (B) REF- or (C) pUL69-interaction. (A) HEK293T cells were either mock transfected (lanes 1 and 2) or cotransfected with plasmids encoding FLAG-tagged UAP56 and myc-tagged REF (lane 3). The expression of endogenous proteins and proteins after transfection was controlled by Western blot analysis using an α-UAP56 antibody (anti-BAT1) (A, upper panel) and an α-REF antibody (A, middle panel). Immunoprecipitation was performed using an α-UAP56 antibody followed by detection of coprecipitated proteins with α-REF antibody (A, lower panel). The position of detected proteins is indicated on the right of each panel (UAP56, FLAG-UAP56, REF, myc-REF). Lysates of lane 1 were treated with RNase in order to exclude a bridging of proteins by RNA. (B) HEK293T cells were transfected with plasmids encoding either myc-tagged-UAP56 variants (lanes 1–9, as indicated) or myc-tagged REF (lanes 10 and 11). The correct expression of proteins after transfection was controlled by Western blot analysis using an α-myc antibody (B, upper panel). Expression of REF (either endogenous or transfected) was also verified by Western blot analysis using an α-REF antibody (B, middle panel). Immunoprecipitation was performed using an α-REF antibody followed by detection of cotransfected proteins with α-myc antibody (B, lower panel). The position of detected proteins is indicated on the right of each panel (Ig_hc_ =  immunoglobulin heavy chain; Ig_lc_ =  immunoglobulin light chain). (C) HEK293T cells were co-transfected with a plasmid encoding pUL69 in combination with vectors coding either for β-galactosidase alone or for fusions of UAP56 with β-galactosidase (as indicated). Expression of β-galactosidase fusion proteins and pUL69 was confirmed by Western blot experiments (C, upper and middle panel, respectively). Immunoprecipitation was performed using an anti-β-galactosidase antibody followed by the detection of coprecipitated proteins using an α-pUL69 antibody (C, lower panel).

Next, the yeast mapping studies had to be corroborated by coimmunoprecipitation experiments in order to exclude that fusion to the GAL4 DNA-binding or GAL4 activation domain affected protein-protein interactions of UAP56/URH49. In a first experiment, we verified that an interaction between endogenous UAP56 and REF can be detected by coimmunoprecipitation, thus confirming the results of previously published studies (Fig. 6A) [34], [35]. Then, in order to delineate the REF interaction region of UAP56, HEK293T cells were transfected with expression plasmids for myc-UAP56-full-length (Fig. 6B, lane 1), N- or C-terminally truncated versions of myc-UAP56 (Fig. 6B, lanes 2 to 9) or myc-REF (Fig. 6B, lanes 10 and 11). Two days later, cells were harvested and protein expression was analyzed by Western blotting (Fig. 6B, upper panel: αmyc; middle panel: αREF). Immunoprecipitation of endogenous REF was performed using an anti-REF antibody (Fig. 6B, lower panel, IP αREF, lanes 1 to 10) or the control antibody anti-myc (Fig. 6B, lower panel, lane 11) and coprecipitated or precipitated proteins were detected by a monoclonal anti-myc antibody (Fig. 6B, lower panel, lanes 1 to 11). As expected from previous experiments, the REF-specific antibody precipitated myc-REF to a similar extent as the anti-Myc antibody (Fig. 6B, lower panel, compare lanes 10 and 11). Importantly, the precipitated endogenous REF protein specifically coprecipitated full-length UAP56 as well as UAP56aa51-428 (Fig. 6B, lower panel, lanes 1 and 2). In contrast, all other N- or C-terminal deletion constructs failed to copurify with REF (Fig. 6B, lanes 3 to 9) although the proteins were properly expressed (Fig. 6 B, upper panel). In summary, we confirmed that the region comprising the amino acids 51–428 of UAP56 are required for interaction with REF.

Finally, we wanted to further narrow down the pUL69-interaction domain within UAP56. As we had reported before that pUL69 is able bind to UAP56aa140-428 in coimmunoprecipitation experiments [26], additional eukaryotic expression plasmids for N-terminally truncated proteins were constructed (UAPaa195-428, UAPaa227-428 and UAPaa298-428). These truncations were cloned in frame into the CFN-βGal vector, thus fusing UAP56 to the heterologous SV40-NLS and to β-galactosidase. This was done in order to ensure a correct nuclear localization of the respective proteins. Furthermore, these constructs could be used in subsequent experiments to delinate the nuclear export signal of UAP56. Then, HEK293T cells were cotransfected with expression plasmids encoding pUL69 either together with a plasmid for βGal fused to the SV40-NLS (NLS-βGal) (Fig. 6C, lane 2) or together with N-terminal deletion constructs of UAP56 fused in frame to βGal-NLS as indicated (Fig. 6C, lanes 3 to 5). Two days later, cells were harvested and protein expression was analyzed by Western blotting using monoclonal antibodies directed against βGal (Fig. 6 C, upper panel) or pUL69 (Fig. 6C, middle panel). When immunoprecipitation was performed using an anti-βGal antibody, only βGAL-NLS-UAP56aa195-428 and –UAP56aa227-428 specifically coprecipitated pUL69 from the lysates (Fig. 6C, lower panel, lanes 3 and 4). These interactions were considered specific as pUL69 was neither coprecipitated by NLS-βGal nor by βGAL-NLS-UAP56aa298-428 (Fig. 6C, lanes 2 and 5) or when no βGAL-protein was present (Fig. 6C, lane 1). In conclusion, this set of experiments confirmed the results obtained in yeast and clearly demonstrates that the residues 227-428 of UAP56 can be tethered to a heterologous protein still preserving their capability to interact with the viral protein pUL69.

### Mapping of the UAP56 nuclear export signal

**Figure 7:**
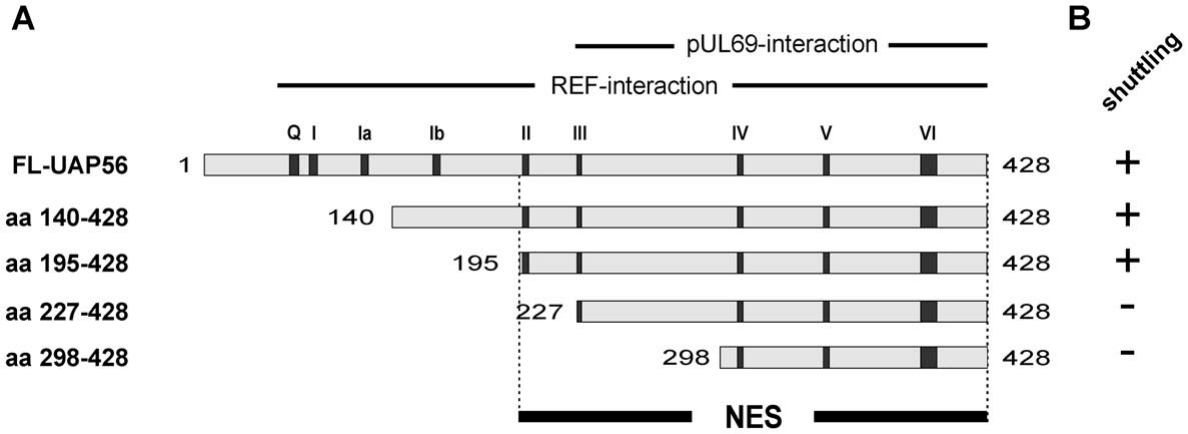
Delineation of the putative NES within the C-terminus of UAP56 by heterokaryon analysis. (A) Deletion mutagenesis of UAP56 was performed to construct the UAP56 mutants indicated in panel A. Highlighted by bars: pUL69-interaction motif (aa227-428); REF-interaction motif (aa81-428); NES, nuclear export signal (aa195-428). (B) The constructs shown in panel A were tested by heterokaryon analyses for their ability to shuttle from the nucleus of a transfected HeLa cell to a murine nucleus. Positive staining of the murine nucleus is indicated by ‘+’, no staining is indicated by a ‘-‘.

Having delineated the domains of UAP56 required for its nuclear localization and interaction with REF or pUL69, we next wanted to further narrow down the motif required for nuclear export. Due to the high homology between URH49 and UAP56, the NES was exemplarily mapped for UAP56. In order to assure the nuclear localization of UAP56 deletion constructs, UAP56aa140-428 was fused to the NLS of the SV40 large T-antigen. As described above, further N-terminal truncations of UAP56 were cloned in frame into the CFN-βGal vector, thereby being tethered to the heterologous SV40-NLS and β-galactosidase. After confirmation of their nuclear distribution by immunofluorescence analyses (data not shown), the indicated constructs (Fig. 7A) were transfected into HeLa cells and assayed in heterokaryon analyses. As controls, HeLa cells were cotransfected with the shuttle-control FLAG-UAP56 or the non-shuttling control IE1p72, respectively. As depicted in figure 7B, UAP56 shuttled via its C-terminus, comprising the amino acids 195-428. In conclusion, our mapping studies revealed that UAP56 can only be marginally truncated without loosing REF-interaction or nuclear localization, while the nucleocytoplasmic shuttling domain can be narrowed down to a smaller C-terminal part of UAP56 comprising its amino acids 195-428 thereby overlapping with its pUL69-interaction motif.

### An N-terminal domain of UAP56 is able to interact with the nuclear pore-associated protein Rae1

**Figure 8:**
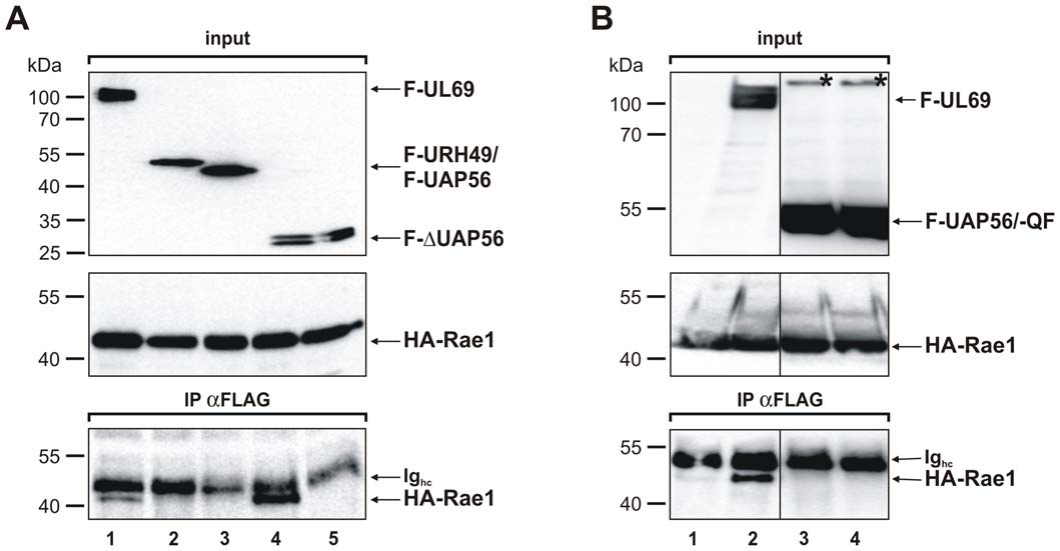
Interaction of Rae 1 with pUL69 and UAP56. (A+B) Coimmunoprecipitation analyses of Rae1 with pUL69 and UAP56/URH49: HEK293T cells were transfected with a vector coding for HA-Rae1 (A, lanes 1-5; B, lanes 1-9) together with FLAG-UL69 (A, lane 1), -URH49 (A, lane 2), -UAP56 (A, lane 3), -UAP56aa1-194 (A, lane 4) or -UAP56aa195-428 (A, lane 5). Alternatively, HA-Rae1 was cotranfected with an empty vector (B, lane 1), FLAG-UL69 (B, lane 2), FLAG-UAP56 wildtype (B, lane 3) or FLAG-UAP56 Q289R+F312A (B, lane 4). Two days posttransfection the amount of protein in the input was analyzed by Western blotting (input, A, lanes 1–5; B, lanes 1–4). Immunoprecipitation was performed using an anti-FLAG antibody and coimmunoprecipitated proteins were visualized by Western blotting using polyclonal HA-antibody (IP αFLAG, A, lanes 1–5; B, lanes 1–4). Asterisks indicate putative dimeric forms of UAP56 and UAP56 Q289R+F312A (B, lanes 3 and 4).

Nucleocytoplasmic shuttling appears to be conserved from yeast to human UAP56, as it has been reported that Uap56p of *Schizosaccharomyces pombe* (*S.pombe)* is also able to exit the nucleus [36]. The NES of Uap56p was mapped to its amino acids 250 to 350, a region that coincided with a domain required for binding of the nuclear pore-associated protein Rae1p. This interaction was critical for both, nucleocytoplasmic shuttling as well as UAP56p-mediated mRNA export [36]. Importantly, two single mutations, Q297R and F320A, within the UAP56p NES-region, partially abolished export, while a combination of both mutations led to a total loss of UAP56p shuttling. Although the overall homology between UAP56/URH49 and their homolog from *S.pombe* is only approximately 70% amino acid identity/78% amino acid similarity, the essential amino acids required for Uap56p shuttling are well conserved in human UAP56 or URH49 and correspond to UAP56 Q289 and F312. Since human Rae1 itself is a known nucleocytoplasmic shuttling protein which exports cellular mRNAs via interaction with the nuclear pore component Nup98 [37], [38], Rae1 seemed to be a promising candidate for the so far unknown export receptor required for CRM1-independent shuttling of human UAP56/URH49. To investigate this, we first examined whether Rae1 is able to interact with UAP56/URH49 in human cells. For this, HEK293T cells were cotransfected with plasmids encoding HA-Rae1 in combination with vectors encoding either full-length and truncated FLAG-tagged UAP56/URH49 or Flag-tagged UL69. As illustrated by Fig. 8A, lower panel, HA-Rae1 was weakly but specifically coprecipitated by FLAG-UL69 (Fig. 8A, lane 1) but neither by full-length UAP56 nor by URH49 (Fig. 8A, lanes 2 and 3). However, the N-terminus of UAP56, comprising amino acids 1-194, was able to coprecipitate Rae1, whereas the C-terminus (aa195-428), required for pUL69-interaction and shuttling, failed to do so (Fig. 8A, compare lanes 4 and 5). This suggests that the Rae1-interaction motif of human UAP56 was different from that detected in *S.pombe.* Since Thakurta and colleagues had demonstrated that two amino acid substitutions within the NES of Uap56p abolished Rae1-binding and shuttling [36], site directed mutagenesis was performed to construct human UAP56, carrying the amino acid substitutions Q289R and F312A (construct named UAP56-QF). After confirmation that UAP56-QF was expressed like wildtype UAP56 in immunofluorescence (data not shown) and Western blot analyses (compare Fig. 8B, input lanes 3 and 4), both constructs were assayed by coimmunoprecipitation for Rae1-interaction. As depicted in Fig. 8B (IP αFLAG, lanes 3 and 4), neither wildtype UAP56 nor UAP56-QF were able to interact with Rae1 while HCMV pUL69 was clearly coprecipitated (Fig. 8B, lane 2, lower panel). In summary, our results suggest that an N-terminal domain of human UAP56 has the capability to bind Rae1 while full-length UAP56 failed to interact.

### Shuttling activity of the mutant UAP56-Q289R+F312A

**Figure 9:**
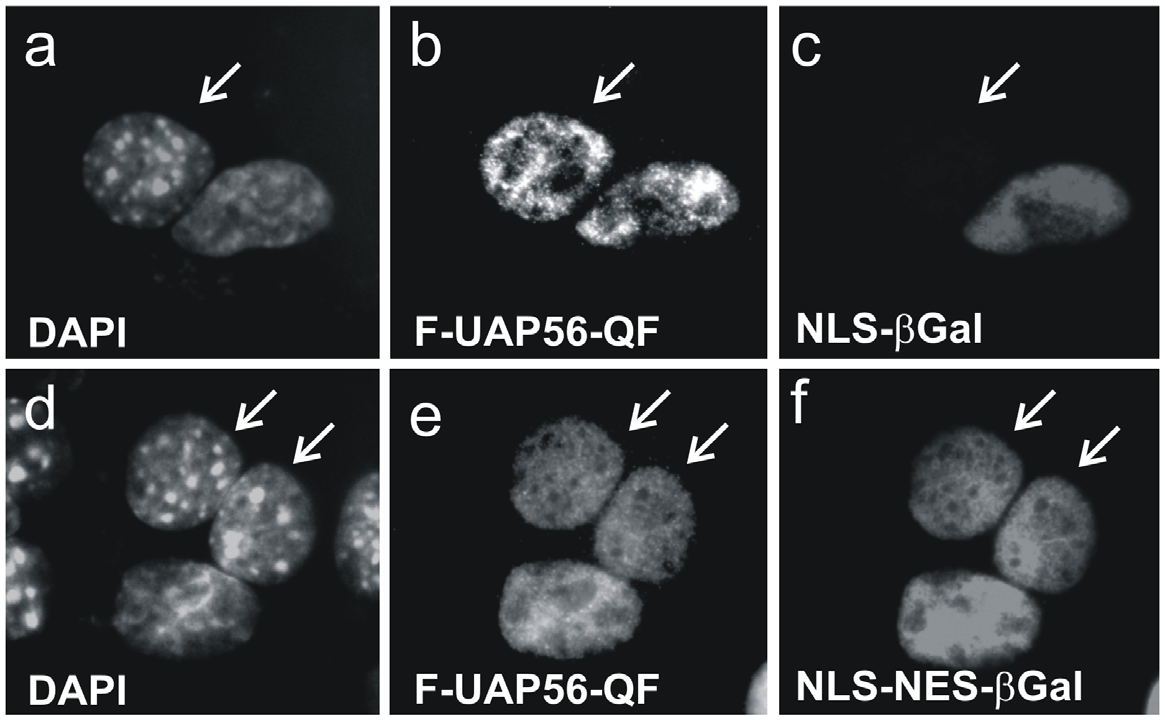
Nucleocytoplasmic shuttling of UAP56-Q289R+F312A. Heterokaryon analysis: HeLa cells were cotransfected with vectors encoding FLAG-UAP56-Q289R+F312A (F-UAP56-QF, panels a-f) and one of the internal control plasmids as indicated (a–c, +CFN-βGal; d–f, +CFNrev-βGal). Transfected cells were subjected to an interspecies heterokaryon assay as described before. Indirect immunofluorescence analysis was performed using polyclonal anti-FLAG serum (b and e) and monoclonal anti-βGal-antibody (c and f). DAPI staining (a and d); murine nuclei are indicated by arrows.

As mentioned before, Uap56p carrying amino acid substitutions Q297R and F320A failed to shuttle in *S.pombe* as well as in human cells [36]. To finally exclude that shuttling of human UAP56 depends on these proposed amino acids, mediating the interaction of UAP56p with Rae1, mutant UAP56-Q289R+F312A was tested in heterokaryon analyses. Hence, HeLa cells were transfected with UAP56-QF (Fig. 9, panels a-f) in combination with either internal control CFN-βGal (panels a-c) or CFNrev-βGal (panels d-f), respectively, and heterokaryon assays were performed. When heterokaryon formation was successful, UAP56-Q289R+F312A (Figure 9, panels b and e) and NLS-NES-βGal (panel f) could be detected in both, murine and human nuclei. In contrast, however, NLS-βGal was detected exclusively in human nuclei (panel c), while UAP56-Q289R+F312A was able to enter the murine nucleus within the same heterokaryon (panel b), hence arguing for the specificity of UAP56-Q289R+F312A shuttling. Thus, while shuttling of yeast Uap56p depends on Rae1p-binding, its human counterpart UAP56 shuttles in a Rae1-independent manner. This finding suggests that, in comparison to Uap56p in *S.pombe,* human UAP56 is a nucleocytoplasmic shuttling protein that is transported via a different export receptor or export pathway.

## Discussion

Although it is well established that the RNA helicases UAP56 and URH49 facilitate the export of mRNAs by a splice- and transcription-coupled recruitment of the adaptor protein REF [12], [15], [23], [39], this study demonstrated for the first time that mammalian UAP56 and URH49 exhibit an intrinsic nucleocytoplasmic shuttling activity, that is CRM1-independent. Our findings suggest an additional cytoplasmic function for UAP56/URH49, an idea that is not without precedent as several other DExD/H-box RNA-helicases involved in mRNA export have been reported to play a dual role both in the nucleus and the cytoplasm. Amongst them, Dbp5, which is known to facilitate mRNA export by remodeling mRNPs at the cytoplasmic filaments of the NPC [40], also functions in translation by promoting stop codon recognition for termination [41]. In analogy, the DEAD-box RNA helicase DDX3, which associates with mRNPs as well as with the export receptor TAP, additionally promotes translation [42]. Moreover, certain shuttling SR proteins, involved in splicing and mRNA-export, exert additional cytoplasmic functions [43], [44]. For example, SF2/ASF promotes the association of spliced and unspliced mRNAs with the translation machinery [45], [46] and SRp20 directly interacts with the cellular internal ribosome entry site binding protein PCBP2 [47]. Thus, our study identified an additional nuclear protein implicated in splicing and mRNA export, being able to shuttle between the nucleus and the cytoplasm.

Our mapping studies revealed that nuclear localization of UAP56 was maintained for those constructs containing the amino acids 81-381 while the residues 51–428 were required for REF-interaction. Therefore, it can be excluded that UAP56 targets the nucleus via interaction with the endogenous nuclear protein REF. Since the very N-terminal 50 amino acids are dispensable for nuclear localization of UAP56, it is probable that URH49, showing its highest divergence to UAP56 within its N-terminal 50 amino acids, enters the nucleus via an analogous domain. Nevertheless, such a region would represent an unusually large nuclear localization signal, thereby suggesting that the complex 3D-structure of UAP56 is disrupted when amino acids within the ‘core-region’ comprising the amino acids 81-381 are deleted. Such an assumption is sustained by the crystal structure of UAP56 that reveals a complex structure built from numerous α-helices and β-sheets [48]. Insofar as UAP56 shuttles via its C-terminus (aa195-428), we can additionally exclude that UAP56 leaves the nucleus via interaction with the shuttle protein REF [18].

Intriguingly, UAP56 tolerated an N-terminal truncation up to residue 227, still preserving its interaction capacity with the viral protein pUL69. This observation implies that pUL69 recruits UAP56 (via its C-terminus) in a REF-unbound form but for proper REF-recruitment, UAP56 has to contain its N-terminus. This assumption would perfectly fit to our current model assuming that pUL69 recruits UAP56 and, hence, REF to nascent mRNA in order to facilitate the export of unspliced mRNA [26], [49], [50].

Interestingly, the nuclear export signal of UAP56 was also mapped to its C-terminus comprising the amino acids 195-428, overlapping with the domain that is required for pUL69-interaction. Based on the available crystal structure of UAP56, this deletion of 194 N-terminal amino acids should lead to a disruption of the complex 3D structure [46]. Surprisingly, however, we detected that this truncated domain was still active for nucleocytoplasmic shuttling and for interaction with pUL69, which may suggest that UAP56 can exist in an alternative conformation in solution. The importance of the nuclear export domain of UAP56 (aa195-428) identified in this study is sustained by a recent publication which demonstrated that Uap56p of *S.pombe* shuttles via its C-terminal residues 250 to 350 [36]. While Uap56p recruits Rae1p and two amino acid substitutions (Q297R and F320A) within the Uap56p NES abolished both, Rae1p-interaction and shuttling, human wildtype UAP56 did not interact with Rae1 and the corresponding amino acid substitutions Q289 and F312 in UAP56 were not able to abrogate nucleocytoplasmic shuttling. These findings suggest that UAP56 (and, hence, most probably URH49) shuttle via an export receptor that is distinct from CRM1 and Rae1.

Although full-length UAP56 was unable to interact with Rae1, surprisingly, an N-terminal fragment comprising the residues 1-194 was capable to do so. The biological significance of this observation is presently not clear. It will require further investigations in order to clarify whether UAP56 contains a cryptic interaction domain for Rae1 that may be activated upon binding to specific cellular proteins. The unexpected finding that full-length UAP56 failed to interact with Rae1 might be explained by the fact that Uap56p of *S.pombe* differs in several properties from its homologs of other species. Similar to UAP56/Sub2p, Uap56p was found to be essential for growth and mRNA export but, in contrast, it is not required for splicing [13], [36], [51]. In addition, Uap56p has a more critical role for mRNA export of *S.pombe* since there, Mex67p (TAP) is dispensable for mRNA export which contrasts to *S.cerevisiae*, fruitfly, nematode or human [52]–[56]. Consistently, our study identified additional differences between UAP56 and Uap56p. While Uap56p contains a bipartite NLS between residues 1-100 and 216-250, human UAP56 tolerated only a truncation of the very N- and/or C-terminal amino acids without loosing nuclear localization, suggesting that human UAP56 has a more complex secondary structure required for nuclear import or, alternatively, that UAP56 might gain access to the nucleus by recruitment of another nuclear interaction protein. Conclusively, while *S.pombe* Uap56p and its human counterpart UAP56 share a well conserved nucleocytoplasmic shuttling activity, their shuttle receptor seems to have diverged during evolution of yeast and human.

Furthermore, our studies identified an interaction of the viral protein pUL69 and the nuclear pore-associated protein Rae1. Since Rae1-interaction with wildtype pUL69 was weak, it could not be excluded that Rae1 might have been bridged via other copurified components of the cellular mRNA export machinery. However, an indirect interaction via copurified RNA was excluded since pUL69aa380-744, deleted of its RNA-binding domain [57], still interacted with Rae1 (data not shown). Although N-terminally truncated versions of pUL69, lacking their UAP56-interaction motif [26], remained residual binding, robust Rae1 association required an intact UAP56- and/or RNA-interaction motif of pUL69 (data not shown), thereby suggesting that pUL69 recruits preformed UAP56- and Rae1-containing complexes and we are currently investigating whether this interaction has an impact on the replication of human cytomegalovirus.

While our results clearly demonstrated nucleocytoplasmic shuttling of mammalian UAP56 or URH49, the functional impact of this capacity remains to be investigated. Besides their role for mRNA export, there is increasing evidence to suggest an involvement of UAP56 and URH49 in other cellular processes. For instance, it was shown that UAP56 and URH49 interact with the nuclear cytokine-induced protein CIP29 (alternatively named HCC-1), that is implicated in the regulation of cell-cycle progression [58]–[60]. Most importantly, however, a recent study demonstrated that, in *Drosophila,* UAP56 executes two independent functions: while its main nuclear function is the well-characterized stimulation of bulk nuclear mRNA export, UAP56 is also required for efficient active mRNA transport in the cytoplasm [61]. Consistent with such a scenario *Drosophila* UAP56 could be detected both in the nucleus and the cytoplasm of *Drosophila* oocytes and it is proposed that UAP56 remains on the RNA after nuclear export and has a role in the remodeling of RNP complexes in the cytoplasm [61]. Thus, it is highly tempting to speculate that the nucleocytoplasmic shuttling that we characterized in this study may be required for a similar activity of mammalian UAP56 for remodeling of RNP complexes in the cytoplasm.

In summary, our study demonstrates for the first time that the mammalian DExD/H-box RNA-helicases UAP56 and URH49 exhibit a CRM1- and Rae1-independent nucleocytoplasmic shuttling activity, thereby suggesting a novel, so far uncharacterized cytoplasmic function of these two RNA-helicases.

## Materials and Methods

### Oligonucleotides and plasmids

Oligonucleotide primers used for this study are listed in Table 1 and were purchased from Biomers GmbH (Ulm, Germany). FLAG-UAP56 was a kind gift of K. Nagata [32], myc-Aly was provided by A. Whitehouse [62] and HA-Rae1 was obtained from B.M. Fontoura [63]. The construction of pHM160, FLAG-UL69, FLAG-UL69-PP602/603AA, FLAG-URH49, myc-pUL84, myc-UAP56, myc-UAP56 aa140-428, myc-URH49, myc-URH49 aa168-427 used for this study was described previously as it was for the corresponding yeast expression constructs [26], [33], [64]. The N- or C-terminally truncated derivatives UAP56 aa51-428, aa81-428, aa195-428, aa227-428, aa311-428, aa1-201, aa1-310 and aa1-381 were constructed via PCR using the respective primer pairs (Table 1), followed by cleavage of the products with *Bam*HI and *Xho*I and subcloning into the Myc-expressing vector pHM1580 [65]. For the generation of Myc-βGAL-deltaUAP56 constructs, β-Galactosidase was amplified from the pSV-βGAL vector (Promega) using the respective primer pair (Table 1). The PCR-product was then digested by *Bgl*II and subcloned in frame into the *Bam*HI-linearized vectors encoding the respective Myc-deltaUAP56 as described above. N-terminal deletion mutants of pUL69 (i.e. encoding amino acids 177-744, 380-744 and 1-630) were constructed analogously to FLAG-UL69 aa92-744 [26] via ligation of the *Bam*HI/*Eco*RV-fragments into pHM972 [66], coding for an N-terminal in-frame fusion of a FLAG tag as well as the simian virus 40 (SV40) T antigen nuclear localization signal (NLS). FLAG-NLS-UAP56 aa140-428 was constructed likewise, albeit a *Bam*HI/*Xho*I PCR fragment was inserted into pHM972. Mutagenesis within FLAG-UAP56 was performed using the Quick Change site-directed mutagenesis kit as instructed by the manufacturer (Stratagene) and a complementary primer pair (as indicated in Table 1), resulting in the plasmid FLAG-UAP56-Q289R+F312A. N- or C-terminal deletion mutants fused to the GAL4-AD (see Fig. 5A) were constructed by inserting *Bam*HI/*Pst*I fragments of UAP56 or *Eco*RI/*Sal*I fragments of URH49 into the pGAD424 vector (Clontech). REF-BD was constructed by digestion of Aly136, which was a kind gift of B. Clements [67] using *Sma*I/*Xho*I and subsequent ligation into *Sm*aI/*Sal*I-digested pGBT9 (Clontech). To tether the UAP56 truncations aa195-428, aa227-428 and aa298-428 to β-galactosidase and the SV40-NLS, the respective PCR fragments were subloned via *Xba*I/*Kpn*I into pCFN-βGal [27].

**Table 1:** Oligonucleotides that were used in this study; restriction sites are underlined.

Designation	Sequence
5UAP-BamH1	GATCGGATCCCCATGGCAGAGAACGATGTGGACAATGAG
5UAPaa51-BamH1	GCATGGATCCGTCTGCTCAAGCCAGAGTTGCTC
5UAPaa81-BamH1	GCATGGATCCGTCTGGGAATGGATGTCCTGTGC
5UAPaa151-BamH1	GCATGGATCCGTGGTCTGTCTATCAAGAAGGAT
5UAPaa195-BamH1	GCATGGATCCGTTTGGATGAATGTGATAAGATG
5UAPaa201-BamH1	GCATGGATCCGTATGCTTGAACAGCTCGACATG
5UAPaa227-BamH1	GCATGGATCCGTTTCAGTGCTACCTTGAGAAAG
3UAP-aa381-Pst1	GCATCTGCAGAAACCGGCCTGCTCTGGCCAC
3UAP-aa400-Pst1	GCATCTGCAGGAGGATCTTGGCATCATTCTC
3UAP-PST	GATCCTGCAGCTACCGTGTCTGTTCAATGTAGGAGGA
5URH-EcoR1	GCATGAATTCATGGCAGAACAGGATGTGGAA
5URHaa50-EcoR1	GCATGAATTCCTGCTGAAGCCGGAGCTCCTG
5URHaa80-EcoR1	GCATGAATTCCTGGGCATGGACGTCCTGTGC
5URHaa150-EcoR1	GCATGAATTCGGTCTCTCCATCAAGAAGGAT
5URHaa194-EcoR1	GCATGAATTCCTGGACGAGTGTGACAAGATG
5URHaa200-EcoR1	GCATGAATTCATGCTGGAGCAGCTGGACATG
5URHaa226-EcoR1	GCATGAATTCTTCAGCGCCACCCTGAGCAAG
3URH-aa380 Sal1	GCATGTCGACAAAGCGACCCGCCCGGGCCAC
3URH-aa399-Sal1	GCATGTCGACGAGGATTTTGGCATCATTCTC
3URH-Sal1	GCATGTCGACTTACCGGCTCTGCTCGATGTA
5mycUAP-BamH1	GCATGGATCCATGGCAGAGAACGATGTGGACAATGAG
5mycUAPaa51-Bam	GCATGGATCCCTGCTCAAGCCAGAGTTGCTC
5mycUAPaa81-Bam	GCATGGATCCCTGGGAATGGATGTCCTGTGC
5mycUAPaa140-Bam	GCATGGATCCATGCCCAATGTCAAGGTTGCTGTT
5mycUAPaa140-NLS-Bam	GCATGGATCCCCATGCCCAATGTCAAGG
5mycUAPaa195-Bam	GCATGGATCCTTGGATGAATGTGATAAGATG
5UAPaa311-Bam	GCATGGATCCAACTTCCCAGCCATTGCCATCC
3mycUAP-aa201-Xho	GCATCTCGAGCATCTTATCACATTCATCC
3mycUAP-aa310Xho	GCATCTCGAGCTGCTCCACTAGTAGCTGG
3mycUAP-aa381Xho	GCATCTCGAGAAACCGGCCTGCTCTGGCCAC
5bGAL-BglII	GCATAGATCTATGCCTTCTGAACAATGGAAAGGC
3bGAL-Bam,BglII	GCATAGATCTGCTAGCGGATCCGGGCCCGGGTTTTTGACACCAGACCAACTGGTAATGG
5UAPaa195Xba1	GATCTCTAGATTGGATGAATGTGATAAGATG
5UAPaa227Xba1	GATCTCTAGATTCAGTGCTACCTTGAGCAAAG
5UAPaa298Xba1	GCATTCTAGACAGCGGTGCATTGCCTTGG
3UAP56w/oStopKpn1	GATCGGTACCACCGTGTCTGTTCAATGTAGGAG
C-UAP-Q289R	GTCCTTGAGTTCAACCGGGTGGTGATCTTTGTG
NC-UAP-Q289R	CACAAAGATCACCACCCGGTTGAACTCAAGGAC
C-UAP-F312A	CTAGTGGAGCAGAACGCCCCAGCCATTGCCATC
NC-UAP-F312A	GATGGCAATGGCTGGGGCGTTCTGCTCCACTAG

### Yeast-transformation and two-hybrid analyses

Yeast cells were transformed using a modified lithium acetate method [68]. For this, yeast of the tryptophane- and leucine-auxotrophous strain Y153 were grown in 6 ml YAPD medium at 30°C overnight. The next day, transformation was prepared by mixing 2 µg of each plasmid with 10 µl of salmon testes carrier DNA [10mg/ml]. After pelleting yeast cells for 2 minutes at 2500 rpm, the supernatant was discarded and the pellet resuspended in 3 ml LP-mix by vortexing after the addition of 300 µl DMSO. 500 µl each of these yeast cells were then resuspended with the DNA, incubated for 15 min at room temperature and subsequently heat shocked for 15 minutes at 42°C. For dilution of the LP-mix, 500 µl sterile H_2_O were added before the cells were pelleted for 2 minutes at 2500 rpm. Thereafter, the supernatant was discarded and the cells were washed again in 1 ml H_2_O. After another centrifugation step the pellet of transformed yeast cells was carefully resuspended in 100 µl of sterile H_2_O and plated on minimal medium agar plates for selection. The plates were incubated at 30°C for appoximately 3 days until colonies appeared.

The activity of the reporter gene β-galactosidase, stably integrated in yeast strain Y153, was examined by filter-lift tests [69]. Therefore, growth selected yeast cells were transferred onto a nylon membrane (Hybond™-C-Extra, GE Healthcare, Freiburg, Germany) and subsequently permeabilized by incubation for one minute in liquid nitrogen. Thereafter, the membrane was put on a substrate-soaked Whatman paper (Buffer Z, 1 mM β-mercaptoethanol, 1.5 mM X-Gal) and incubated at 30°C for 4–12 hours. Protein-protein interactions were indicated by blue staining of the respective yeast cells.

### Cell culture and plasmid transfections

HEK293T cells were cultivated in Dulbecco's modified Eagle medium (DMEM) containing 10% fetal calf serum, NIH3T3 cells were cultivated in DMEM containing 8% fetal calf serum and HeLa cells were cultivated in Eagle's minimal essential medium with 5% fetal calf serum. Transfection of HEK293T cells was performed at a cell confluency of app. 80% via precipitation of calcium phosphate-DNA complexes as described earlier [65], [70]. Lipofectamine 2000 (Invitrogen) was applied according to the protocol of the manufacturer to transfect HeLa cells at a confluency of 70%.

### Coimmunoprecipitation assay (CoIP)

Coimmunoprecipitation analysis was performed as decribed by Bannister and Kouzarides [71]. Briefly, HEK293T cells were transfected in 6-well plates (5.0×10^5^ cells/well). Two days post transfection, cells were lysed in 800 µl of CoIP buffer (50 mM Tris-HCl pH 8.0, 150 mM NaCl, 5 mM EDTA, 0.5% NP-40, 1 mM PMSF, 2 µg/ml aprotinin, 2 µg/ml leupeptin and 2 µg/ml pepstatin) and used for CoIP as described before [33]. The precipitates were subjected to a standard Western blot (Wb) analysis using specific antibodies (MAb-FLAG M2, Sigma; PAb-HA, Covance, CA, USA; MAb-Myc 1-9E10.2, ATCC; anti-Aly [11G5], Abcam; anti-β-galactosidase, Millipore, anti-BAT1, Proteintech Group, and MAb-UL69 69-66) for precipitation or for the detection of coimmunoprecipitates (ECL staining, New England Bio-Labs).

### Indirect immmunofluorescence analysis

HeLa cells were grown on coverslips in 6-well dishes (3.0×10^5^ cells/well) and transfected by standard calcium phosphate precipitation one day after seeding. Two days later, cells were fixed with 4% paraformaldehyde (10 min, room temperature) and permeabilized using PBS/0.2% Triton-X-100 (20 min, 4°C). After incubation with the appropriate primary antibody (MAb-Myc 1-9E10.2, ATCC, US; MAb-β-Gal, Roche, Germany) for 45 min at 37°C, cells were excessively washed and subsequently incubated for 30 min at 37°C with FITC- and/or Cy3-conjugated secondary antibodies (Dianova, Germany); counterstaining of the cell nuclei was achieved by DAPI Vectashield mounting medium (Vector Laboratories, US). Immunofluorescence data were analyzed by an Axiovert-135 microscope at magnifications of 400x and 630x (Zeiss) [65].

### Nucleocytoplasmic shuttling assay

To examine the nucleocytoplasmic shuttling activity of UAP56/URH49, interspecies heterokaryon analyses were performed [72]. After fusion of transfected HeLa cells with non-transfected murine NIH3T3 cells by PEG3500, proteins were allowed to shuttle for 4 hours exactly as described by Lischka and colleagues [64]. When inhibition of CRM1-dependent export was required, cells were treated with 2.5 ng/ml LMB starting 3h prior and throughout the whole experiment. Cells were fixed by 4% paraformaldehyde and heterokaryons subjected to standard indirect immunofluorescence analyses as described above, using mouse-anti-β-galactosidase antibody (MAb-β-gal, Roche, Germany) and FLAG-tag specific antibody (RAb-FLAG, Sigma, Germany) as primary antibodies.

### *In silico* analyses

For prediction of classical NLS and NES sequences we took advantage of the software provided by http://cubic.bioc.columbia.edu/services/predictNLS and www.cbs.dtu.dk/services/, *respectively.*
